# Pediatric Herpes Zoster

**DOI:** 10.5811/cpcem.2019.10.44301

**Published:** 2019-12-17

**Authors:** Daniel Quesada, Larissa Morsky, Phillip Aguìñiga-Navarrete, Madison B. Garrett

**Affiliations:** * LAC+USC Medical Center, Department of Emergency Medicine, Los Angeles, California; † Kern Medical, Department of Emergency Medicine, Bakersfield, California

## Abstract

A 10-year-old male vaccinated against varicella had developed left-sided rashes on his thoracic region in single dermatomal distribution, which is consistent with herpes zoster. Although herpes zoster is uncommon in children, especially with the current vaccination regimen, this case report serves as a reminder to consider it in one’s differential diagnoses, even in the immunocompetent, fully immunized pediatric patient. This is a case report of a previously healthy, fully vaccinated child who developed herpes zoster.

## INTRODUCTION

The varicella vaccination was introduced in 1995 as a one-time shot to be given at 12–18 months.^1^ The current Centers for Disease Control and Prevention recommendations are a two-shot series given between 12–15 months and again at four-six years.^2^ The duration of immune-protection ranges from 6–20 years.^2^ One study demonstrated a decrease in risk of four to 12 times in vaccinated children under 10.^1^ The vaccine has been proven beneficial in largely preventing herpes zoster (HZ) and blunting the course of the infection. It was reported that children who were vaccinated and presented with HZ were likely to be less than 10, have lower associated pain, and a zoster rash in the lumbosacral region with fewer vesicles that were smaller in size.^1^

There has been a reported higher incidence of HZ in children vaccinated after the age of five as compared to those vaccinated between 12–18 months.^3^ A retrospective study demonstrated a 2.1-year average incidence of HZ after vaccination.^3^ Prior to vaccination, the incidence of HZ was 46 per 100,000 cases for children under 14.^3^ With the increase in the number of shots from 1995 to today, there has been a decreased incidence of HZ from 2003 to 2014, 74 per 100,000 person years for unvaccinated children and 38 per 100,000 person years for children who are vaccinated.^4^

One case-control study identified race as a risk factor for vaccinated children developing HZ. Black children were at a lower risk than White or Asian.^5^ Incidents were found to be highest in White non-Hispanic children followed by Hispanic children.^1^ Retrospective studies also demonstrated that children vaccinated at one to two years had an increased chance of HZ when compared to their unvaccinated counterparts.^4^ Although there have been cases of HZ caused by wild-type virus and vaccine-strain virus in children who have been vaccinated, there is still consistently strong clinical support for the routine use of the varicella vaccine. Even in vaccinated children, wild-type virus was more likely to be isolated than the vaccine strain.^3^, ^5^–^11^ With the changes in the shot series, there has been a decrease in the risk of wild-type virus causing HZ.^3^

## CASE REPORT

A previously healthy 10-year-old Hispanic male presented with a chief complaint of a rash on his left chest and back for three days. The rash began gradually, which was described as both itching and burning in sensation. Prior to the onset of the rash, the patient stated that he had felt a burning sensation to the area where the rash eventually developed. According to his parents, the patient was fully vaccinated, including the varicella vaccine, and had never contracted chicken pox. They denied any sick contacts at home. The patient had no other known risk factors such as trauma, family history of HZ, autoimmune diseases, or other malignancies that would have predisposed him to HZ. All reviews of systems were negative.

Physical examination was remarkable for grouped vesicles noted on the left anterior chest and healing vesicles with eschars noted on the left upper back (Image). The rash appeared along the sixth thoracic dermatome. The rash was painful, blanched with palpation, and was without induration or discharge. Vital signs and the remainder of the physical examination were unremarkable. Diagnosis of HZ was clinical, given the classic unilateral dermatomal distribution and vesicular appearance of the rash. The patient remained stable during his emergency department (ED) visit and was discharged home with a prescription for acyclovir and acetaminophen for anticipated neuropathic pain, generally associated with HZ. Patient was advised to follow up with his pediatrician in one week.

The patient’s mother was contacted approximately three weeks after his ED visit using a Spanish-speaking phone translator. The mother informed the author that the rash had lasted approximately eight days and the lesions were completely healed the following week. She reported that he no longer had any pain at the site of the rash. Of note, his younger sister, who was 10 months old, developed a rash all over her body, including back, buttocks, and legs, and was diagnosed with varicella.

## DISCUSSION

Herpes zoster, also known as shingles, is a condition caused by reactivation of the varicella zoster virus, which causes varicella or chicken pox, the result of primary infection by the virus. Although HZ incidence has significantly fallen since the introduction of the live-attenuated varicella vaccination in 1995 to prevent primary infection, varicella primarily affects children and is self-limited. While vaccinated children do not present with varicella, both vaccine-type and wild-type viruses remain dormant in sensory ganglia and can be reactivated, usually during times of stress, such as in trauma or malignancy.

HZ is typically seen in older individuals or those with decreased cell-mediated immunity. When infection occurs in younger age groups, it tends to be less severe. The reactivation typically presents initially with a prodrome of pain and paresthesia, followed by a macular rash. After approximately 24 hours, a painful vesicular rash erupts, confines to one dermatome, and eventually ruptures, crusts, and then resolves. Itching, pain and paresthesia persist throughout the disease course and can be described as having a burning, sharp and lancinating sensation. There can also be associated hyperesthesia and hyperalgesia. Post-herpetic neuralgia can follow after a resolved outbreak and can persist from a few months to indefinitely.^12^ Ocular complications can also occur in patients with involvement of the ophthalmic division of the trigeminal nerve.

**CPC-EM Capsule**

What do we already know about this clinical entity?

Herpes Zoster is uncommon in children after the advent of the Varicella vaccine in 1995 and current CDC prevention recommendations of a two-vaccine series.

What makes this presentation of disease reportable?

Despite being fully vaccinated and otherwise healthy with no prior history of primary varicella infection, a child may develop Herpes Zoster secondary to Varicella-zoster virus reactivation.

What is the major learning point?

It is important for the clinician to keep the differential broad when evaluating pediatric rashes despite vaccine history, age, or absence of history of primary varicella infection.

How might this improve emergency medicine practice?

The ability to diagnose this disease process in a timely manner facilitates the implementation of immediate treatment which may shorten the course of illness and reduce post herpetic sequelae.

Treatment typically consists of antiviral agents such as oral acyclovir if received within 72 hours after the onset of a rash, which decrease the duration of the rash and severity of pain.^13^ The utility of antiviral therapy on patients presenting beyond the 72-hour period has not been amply studied. However, despite insufficient evidence, antiviral therapy is recommended for patients presenting 72 hours after the onset of rashes, including new vesicle formation, ocular, neurologic or cutaneous compilations, and immunocompromise.^14^ While famciclovir and valacyclovir are preferred due to simpler dosing schedules and pharmacokinetic characteristics, patients should be treated with acyclovir if neither are available.^14^

## CONCLUSION

Herpes zoster incidence has become increasingly uncommon in EDs of developed nations with the advent of the varicella vaccine, but it can still occur and should be considered in an emergency physician’s differential even in immunocompetent, fully vaccinated children.

## Figures and Tables

**Image:**
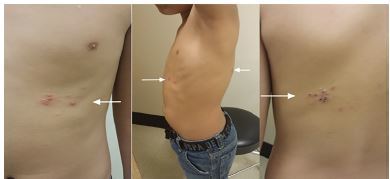
Vesicular rash in the left thoracic region distributed in single dermatome consistent with herpes zoster (arrows).
